# Exploring the Laplace Prior in Radio Tomographic Imaging with Sparse Bayesian Learning towards the Robustness to Multipath Fading

**DOI:** 10.3390/s19235126

**Published:** 2019-11-22

**Authors:** Zhen Wang, Xuemei Guo, Guoli Wang

**Affiliations:** 1 School of Electronics and Information Engineering, Sun Yat-sen University, Guangzhou 510006, China; 2 School of Data and Computer Science, Sun Yat-sen University, Guangzhou 510006, China; guoxuem@mail.sysu.edu.cn; 3 Key Laboratory of Machine Intelligence and Advanced Computing, Ministry of Education, Guangzhou 510006, China

**Keywords:** radio tomographic imaging, RF sensor, received signal strength, laplace prior, multipath fading

## Abstract

Radio tomographic imaging (RTI) is a technology for target localization by using radio frequency (RF) sensors in a wireless network. The change of the attenuation field caused by the target is represented by a shadowing image, which is then used to estimate the target’s position. The shadowing image can be reconstructed from the variation of the received signal strength (RSS) in the wireless network. However, due to the interference from multi-path fading, not all the RSS variations are reliable. If the unreliable RSS variations are used for image reconstruction, some artifacts will appear in the shadowing image, which may cause the target’s position being wrongly estimated. Due to the sparse property of the shadowing image, sparse Bayesian learning (SBL) can be employed for signal reconstruction. Aiming at enhancing the robustness to multipath fading, this paper explores the Laplace prior to characterize the shadowing image under the framework of SBL. Bayesian modeling, Bayesian inference and the fast algorithm are presented to achieve the maximum-a-posterior (MAP) solution. Finally, imaging, localization and tracking experiments from three different scenarios are conducted to validate the robustness to multipath fading. Meanwhile, the improved computational efficiency of using Laplace prior is validated in the localization-time experiment as well.

## 1. Introduction

Target localization has many applications in practice, such as terrorist localization for military and police surveillance [1,2], intruder detection [3], traffic flow and roadside monitoring [4], residential monitoring [5,6] and ambient assisted living [7,8] etc. There are many types of sensors that can be employed for target localization, such as optical cameras [9], ultrasonic sensors [10], light sensors [11], pyroelectric infrared (PIR) sensors [12,13] etc. Among them, RF sensors have the advantages of penetrating walls, smoke and other opaque obstructions, rendering it more appropriate for working in obstructed environments [14]. For RF sensing, narrow-band RF sensing strategy can locate the target by using the ubiquitous and inexpensive wireless devices compared with ultra-wide band (UWB) [1]. This advantage facilities the deployment of dense wireless networks for target localization.

In narrow-band RF sensing, received signal strength (RSS) and channel state information (CSI) are two types of information that can be used for target localization, but the CSI information is obtained by adopting special Wifi network interface card [15,16], which adds additional hardware cost for the localization system. This paper focuses on the low-cost localization system by utilizing only the RSS information [1,17]. Currently, there are four widely used approaches to relate RSS measurements with the target’s location: particle filter method [18,19], fingerprint method [20,21], geometric method [22,23] and radio tomographic imaging [1,2,3,4,7,14,24,25,26,27]. This paper focuses on RTI method.

RTI was firstly proposed by Wilson and Patwari in [1]. When a target enters or moves into the area covered by a wireless network, it will block the line-of-sight (LOS) of some RF links in the wireless network. Thus the RSS measurements on these links will decrease due to the shadowing effect. The shadowing effect is characterized by a shadowing image in RTI. Different from conventional camera images, the pixel value in the shadowing image quantifies how much change of attenuation is experienced at each pixel after the target’s presence. Usually, large pixel values appear at few pixels near the position where the target stands. While the pixels that are far from the target should have little or zero pixel values. Therefore, the shadowing image can be considered as sparse signal and the task of RTI is to reconstruct the sparse shadowing image from the variations of RSS measurements. After that, the target’s position is estimated at the pixel with maximum pixel value in the shadowing image.

However, because of the multi-path interference, it is quite common to observe that the RSS variations may show increase or remain unchanged when the links are blocked by the target. Meanwhile, RSS measurements from links that are not blocked by the target may show decreases because of the fading from non-line-of-sight (NLOS) paths [28,29,30]. If these unreliable RSS measurements are used for image reconstruction, some artifacts (Artifact is defined as a pixel that shows non-zero pixel-value but without a target standing there) will appear in the shadowing image [1], and thereafter, the target’s position may be wrongly estimated because of the artifacts. Thus, how to guarantee the image quality in the case of multipath interference is pretty important.

One approach in enhancing the robustness to multipath fading is to increase the number of informative shadowing RSS measurements by adopting various strategies. The first strategy is from the aspect of antenna. The work in [31] employs unidirectional antennas to replace omnidirectional antennas to enhance the quality of RSS measurements. Also, the specifically designed E-shape antenna [32] and multiple antennas with spatial diversity [33] are implemented to reduce the interference from NLOS multipath fading. However, the specifically designed antenna increases the hardware cost of the RTI system. The second strategy is by virtue of transmitting at multiple frequencies [34,35,36] and multiple transmitting-power levels [37] to obtain more reliable RSS measurements based on off-the-shelf low-cost RF node. In this strategy, fade level plays an important role in evaluating whether the RSS measurement is informative or not [38]. Inevitably, this strategy increases the communication latency and power consumption of the wireless network, which is not appropriate for the real-time communication and battery powered RF node.

Another approach in alleviating the interference from multipath fading lies in software by developing more efficient and robust reconstruction algorithms. The work in [1,14] adopts Tikhonov regularization method to obtain a smooth shadowing image, which could reduce the isolated artifacts caused by multipath fading. However, there are still some noisy block artifacts in the shadowing image which may render the target’s position wrongly estimated. Based on the multi scale model [39], a back-projection and integrated imaging is used to reduce the multipath fading in [40]. Nevertheless, one drawback which hinders this approach to the practical application is that the parameters in this approach are quite sensitive to the scenario. Namely, this approach is lack of robustness. The work in [41] tries to tackle the multipath interference by separating the attenuation image into two components: the static environmental background image and the dynamic target-induced foreground image. However, to obtain the background image, the SVD (Singular Value Decomposition) operation is introduced at each iteration, which greatly increases the computational complexity. Based on the sparse property of the shadowing image and the non-stationary property of the noise from multipath fading, our previous work [42,43] formulate the heterogeneous Bayesian compressive sensing (HBCS) to improve the robustness to multipath fading. Therein, the *student-t* prior is utilized to describe the shadowing image, and the reconstructed image still contains some artifacts especially in the cluttered environment with rich multipath fading. In addition, the HBCS is computationally intensive. In this paper, the sparsity-promoting Laplace prior is exploited to characterize the shadowing image and the fast algorithm is developed to improve the computational efficiency. The contribution of the proposed method is summarized as follows.

- The image quality and localization accuracy are improved by enhancing the robustness to multipath fading.
- The computational cost in localization becomes reduced as well, this is owning to the adoption of the fast algorithm.

The remainder of this paper is organized as follows. Section 2 gives some preliminary knowledge about RTI and presents the problems that the paper will address. Section 3 describes the proposed method in detail. In Section 4, the experiments are conducted to validate the effectiveness of the proposed approach. Finally, the conclusion is given in Section 5.

## 2. Preliminaries and Problem Statement

### 2.1. Radio Tomographic Imaging

**Figure 1:**
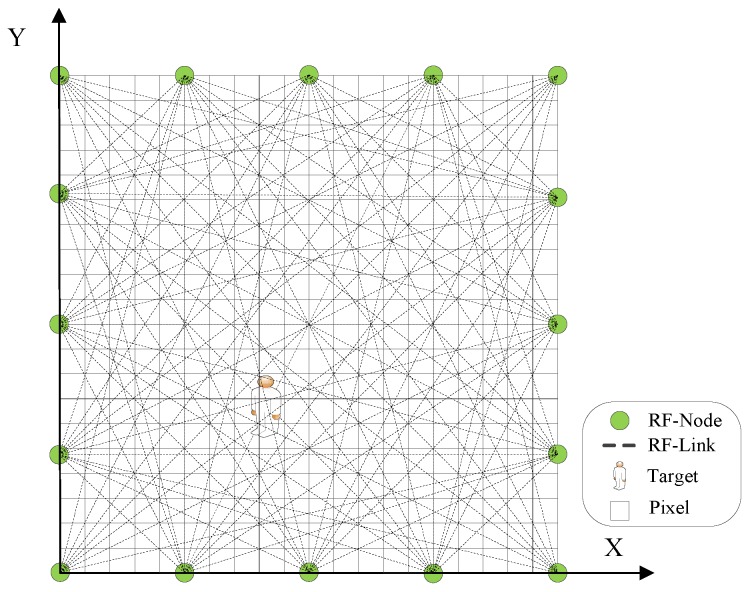
An illustration of the wireless network for RTI.

Before performing RTI, a wireless network consisting of several RF nodes should be deployed firstly in the monitored area. As illustrated in Figure 1, 16 RF nodes build 16*(16–1)/2 = 120 bidirectional RF links. In RTI, the monitored area is considered as an image by dividing into several pixels according to an user-defined pixel-size δ. Moreover, a coordinate system needs to be established to determine the coordinate for each pixel and each RF node.

Usually, a calibration procedure is required to obtain the RSS measurements in the empty scenario, which are denoted by $\bar{r}={\left[{\bar{r}}_{1},{\bar{r}}_{2},\cdots ,{\bar{r}}_{M}\right]}^{T}$, where *M* is the number of bidirectional links in the wireless network. Thereafter, the RSS measurements when the target enters into the area are recorded, which are represented by $r={\left[r_{1},r_{2},\cdots ,r_{M}\right]}^{T}$. RTI takes the RSS variation $y=\bar{r}-r={\left[y_{1},y_{2},\cdots ,y_{M}\right]}^{T}$ as the observation [1]. Seeking for online method to acquire $\bar{r}$ is not the focus of this paper, the readers can refer to [5,6,7,44] and the references therein.

According to the derivation in [1], RTI can be modeled as the following linear equation

(1) y=Φx+n,

where $x={\left[x_{1},x_{2},\cdots ,x_{N}\right]}^{T}$ represents the vector of the shadowing image, denoting the variation of the attenuation image (also called shadowing image) between the empty scenario and the scenario after the target’s presence. The pixel value in the shadowing image can indicate where the target locates. Here *N* equals to the number of pixels. Usually, large pixel values occur near the position where the target locates.

$n={\left[n_{1},n_{2},\cdots ,n_{M}\right]}^{T}$ is the measurement noise due to interference from multi-path fading and sensor accuracy. $\Phi \in R^{M\times N}$ is the measurement matrix determined by the measurement model. In this paper, the ellipse measurement model [1] is adopted, which is expressed as follows

(2) $$\phi_{i,j}=\frac{1}{\sqrt{dis}}\left\{\begin{array}{cc} 1 & if\;d_{i,j}^{TX}+d_{i,j}^{RX}<dis+\eta \\ 0 & otherwise \end{array}\right.,$$

where dis is the distance between the transmitter node and receiver node, $d_{i,j}^{TX}$ and $d_{i,j}^{RX}$ denote the distances from the centre of the *j*-th pixel to the transmitter and receiver of the *i*-th link, and η is a tunable parameter characterizing the width of the ellipse.

In RTI, the target’s position needs to be estimated from the shadowing image, thus the first step of target localization is to reconstruct the shadowing image x from the RSS variation y. After the image vector x is obtained, it is re-arranged to the format of 2D image colum by colum, and the target is then located at the coordinate of the pixel with maximum pixel value. Therefore, the main task of RTI is to reconstruct the shadowing image x from the RSS variation y.

### 2.2. Problem Statement

**Figure 2:**
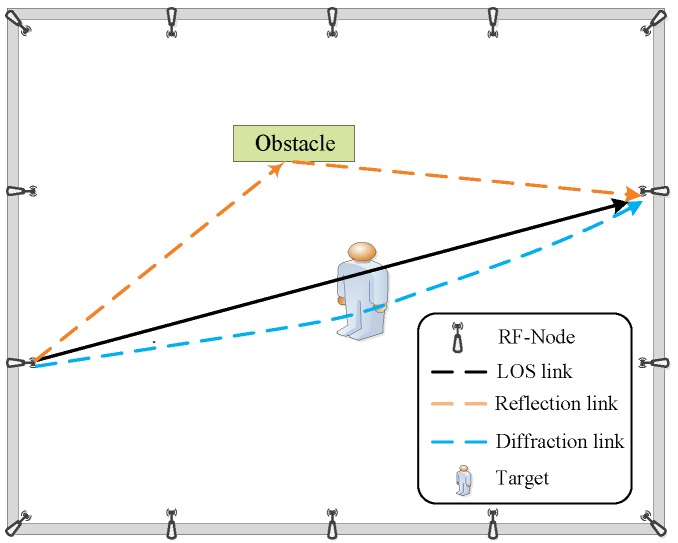
LOS path and NLOS paths from reflection and diffraction.

The most challenging problem in RTI is that not all the RSS variations (i.e., $y=\bar{r}-r$) are reliable due to multi-path fading [1,17,45]. Specifically, because the RSS measurement from the narrow-band RF sensor is a mixed RSS from both the LOS and NLOS paths, the RSS variation may not show decrease even if the target shadows the LOS of that link. The NLOS RSS components from reflection and diffraction caused by the target may overwhelm the LOS component from shadowing effect, thus the RSS variations may increase or remain unchanged. Figure 2 illustrates the LOS path and two NLOS paths from reflection and diffraction.

Furthermore, for the link that is not obstructed by the target, its corresponding RSS measurement may also be possible to show a decrease. This is because the target may introduce reflection on the NLOS path and the reflection could lead to decreased RSS variation because of small-scale fading. This kind of RSS variation is taken as fake-shadowing RSS variation. The more cluttered the environment is, the more severely the multipath fading exhibits on the RSS measurement. If the unreliable RSS variation is used for image reconstruction, some artifacts will emerge in the shadowing image, which will deteriorate the localization accuracy [1,24,41].

As analyzed in the third paragraph of Section 1, the shadowing image is sparse. In dealing with the sparse signal, SBL [46] provides one effective paradigm for the signal recovery due to its sufficient consideration on the signal and noise prior. By dynamically adjusting the objective function in each iteration, SBL could make use of the collected data more effectively. In RTI, it means that SBL could give superior treatment to the informative data (i.e., the RSS variations from shadowing effect) in its dynamic learning process if proper prior is provided in advance [42,43,47]. Therefore, the key problem in applying SBL to RTI is to build appropriate priors for the shadowing image and observed noise.

Existing studies on the application of SBL to RTI put more emphasis on the noise prior [42,43], while for the signal (i.e., the shadowing image), the *student-t* prior is still adopted. One drawback of the *student-t* prior is that it does not produce sufficiently sparse solutions, i.e., the recovered shadowing image is not sparse enough and still contains certain number of artifacts especially in the cluttered environment. Also it is usually computationally intensive. In this paper, to reduce the artifacts in the shadowing image caused by multipath fading, we explore the sparsity-promoting Laplace prior to characterize the shadowing image, and develop the fast algorithm to improve the computational efficiency. Meanwhile, the Jeffrey’s prior is also discussed in this paper.

## 3. RTI using SBL with Laplace Prior

### 3.1. Laplace Prior used to Characterize the Shadowing Image

Under the framework of SBL, all unknowns are taken as random variables with certain probabilistic distributions. Here in RTI, the signal x (i.e., the shadowing image) is constrained by the Laplace prior, which is presented as follows

(3) $$P\left(x|\lambda \right)=\frac{\lambda }{2}\;exp\left\{-\frac{\lambda }{2}\sum_{i=1}^{N}\left|x_{i}\right|\right\},$$

where λ is the scale parameter of the Laplace distribution.

One problem for the Laplace distribution in Equation (3) is that it is not conjugate to the Gaussian distribution, which causes the intractability for Bayesian analysis. To circumvent this problem, inspired by the work in [48], a 3-layer hierarchical prior with similar property is employed to approximate the Laplace distribution. Specifically, a zero-mean Gaussian distribution is imposed on the first layer shown as

(4) $$P\left(x|\gamma \right)=\prod_{i=1}^{N}N\left(x_{i}|0,\gamma_{i}\right),$$

where $\gamma ={\left[\gamma_{1},\gamma_{2},\cdots ,\gamma_{N}\right]}^{T}$. In the second layer, each $\gamma_{i}$ is considered to be i.i.d (independent identically distributed) with the following Gamma distribution

(5) $$P\left(\gamma_{i}|\lambda \right)=Gamma\left(\gamma_{i}|1,\frac{\lambda }{2}\right).$$

Moreover, the third layer constrains λ using a Gamma distribution as well

(6) $$P\left(\lambda \right)=Gamma\left(\lambda |\nu ,\nu \right),$$

where ν denotes the shape and scale parameters of the Gamma distribution, which is defined by the user in practice.

In SBL, the conditional distribution for the RSS variation y also requires to be assigned, here the following Gaussian distribution is given to describe this conditional distribution

(7) $$P\left(y|x;\beta \right)=N\left(y|\Phi x,\beta^{-1}I\right),$$

where I stands for the identity matrix. Based on Equation (7) and y=Φx+n, one can derive that the noise n is indeed assumed to satisfy a zero-mean Gaussian distribution shown as follows

(8) $$P\left(n|\beta \right)=N\left(n|0,\beta^{-1}I\right)=\prod_{i=1}^{M}N\left(n_{i}|0,\beta^{-1}\right),$$

where $\beta^{-1}$ represents the noise variance. To extend the generality of the noise, following the framework of SBL in [46], β is then taken as a random variable and constrained by a Gamma distribution

**Figure 3:**
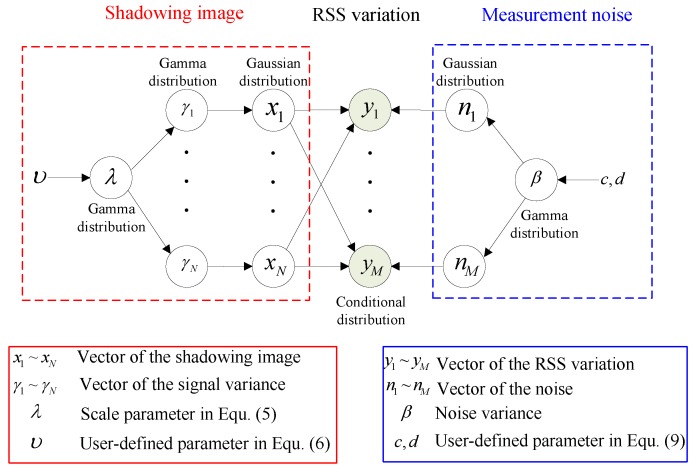
Graph of the hierarchical priors for the signal and noise in RTI.

(9) P(β)=Gamma(β|c,d),

where *c* and *d* are the user-defined parameters, characterizing the shape parameter and scale parameter of the Gamma distribution respectively. To understand the hierarchical priors more vividly, Figure 3 presents the graphical explanation for the signal and noise priors.

In Figure 3, the left side depicts the signal with a 3-layer hierarchical prior, namely Equation (4) to Equation (6). The right side illustrates the noise structure with a 2-layer hierarchical prior, corresponding to Equation (8) to Equation (9). The directional arrow indicates that there is a dependency between two variables. The variable at the end of the arrow is determined by the variable at the start of the arrow.

### 3.2. Bayesian Inference to Obtain the Sparse Maximum-a-Posterior Solution

The Bayesian inference is to seek for the maximum-a-posterior (MAP) sparse estimation about the shadowing image x. The inference is based on the following posterior distribution

(10) $$\begin{array}{cc} P(x|y,\gamma ,\lambda ,\beta ) & =\frac{P(y|x;\beta )P(x|\gamma )P(\gamma |\lambda )P(\lambda )P(\beta )}{P(y,\gamma ,\lambda ,\beta )}\\ \; & ={\left(2\pi \right)}^{-\frac{N}{2}}{\left|\Sigma \right|}^{-\frac{1}{2}}exp\left[-\frac{1}{2}\left(x-\mu \right)\Sigma^{-1}{\left(x-\mu \right)}^{T}\right]. \end{array}$$

As can be seen, the posterior distribution follows a multivariate Gaussian distribution with posterior mean μ and covariance Σ expressed as follows

(11) $$\begin{array}{cc} \mu & =\Sigma \Phi^{T}By \end{array}$$

(12) $$\begin{array}{cc} \Sigma & ={\left[\Phi^{T}B\Phi +A\right]}^{-1}, \end{array}$$

where $A=diag(\gamma_{1},\gamma_{2},\cdots ,\gamma_{N})$, B=βI and I is the *M* x *M* identity matrix. The detailed derivation of Equations (10)–(12) can be found in [46] and the references therein.

γ,λ and β are called hyper-parameters, their optimal solutions are approximated using the type-II maximum likelihood method. In which, the logarithm of the likelihood function is formulated as follows

(13) $$\begin{array}{cc} L(\gamma ,\lambda ,\beta ) & =log\int P(y|x;\beta )P(x|\gamma )P(\gamma |\lambda )P(\lambda )P(\beta )dx\\ \; & =-\frac{1}{2}\left[Mlog\left(2\pi \right)+log|C|+y^{T}C^{-1}y\right]+Nlog\left(\frac{\lambda }{2}\right)+\Sigma_{i=1}^{N}\left(-\frac{\lambda }{2}\gamma_{i}\right)\\ \; & \;\;\;\;\;+(\nu -1)log(\lambda )-\nu \lambda +(c-1)log(\beta )-d\beta , \end{array}$$

where $C=B^{-1}+\Phi A^{-1}\Phi^{T}$. Taking the partial derivatives of Equation (13) with respect to $\gamma_{i},\lambda ,\beta$ and setting them equal to zero, then the optimal solutions are obtained as follows

(14) $$\begin{array}{cc} \gamma_{i} & =-\frac{1}{2\lambda }+\sqrt{\frac{1}{4\lambda^{2}}+\frac{\mu_{i}^{2}+\Sigma_{ii}}{\lambda }} \end{array}$$

(15) $$\begin{array}{cc} \lambda & =\frac{N-1+\nu }{\Sigma_{i=1}^{N}\left(\frac{\gamma_{i}}{2}\right)+\nu } \end{array}$$

(16) $$\begin{array}{cc} \beta & =\frac{N-\Sigma_{i}\left(1-\gamma_{i}^{-1}\Sigma_{ii}\right)+2\left(c-1\right)}{{∥y-\Phi \mu ∥}_{2}^{2}+2d}\;. \end{array}$$

In Equations (14) and (16), $\mu_{i}$ is the *i*-th element of μ, $\Sigma_{ii}$ represents the *i*-th diagonal element in Σ and $\Sigma_{i}$ is the summation notation.

As can be seen from Equations (11) and (12) and Equations (14)–(16), the updates of μ and Σ depend on the hyper-parameters $\gamma_{i},\lambda ,\beta$, meanwhile the updates of $\gamma_{i},\lambda ,\beta$ depend on μ and Σ. Thus an iterative procedure needs to be performed to obtain the solutions. The main computational cost is the matrix inversion in Equation (12), which requires $O(N^{3})$ operation in each iteration [48,49,50], where *N* stands for the total pixel number. The above presented non-fast algorithm is impractical in practice, particularly in the large wireless area where *N* (i.e., the pixel number) is huge.

Here, we present the fast algorithm to improve the computational efficiency. In which, only one $\gamma_{i}$ is updated at each iteration instead of updating the whole vector γ. Following the method in [48,49], the signal hyper-parameter $\gamma_{i}$ is updated as follows

(17) $$\gamma_{i}=\left\{\begin{array}{cc} \frac{-s_{i}\left(s_{i}+2\lambda \right)+s_{i}\sqrt{\Delta }}{2\lambda s_{i}^{2}} & if\;q_{i}^{2}-s_{i}>\lambda \\ 0 & otherwise \end{array}\right.,$$

where $\Delta ={\left(s_{i}+2\lambda \right)}^{2}-4\lambda \left(s_{i}-q_{i}^{2}+\lambda \right)$ with $s_{i}$ (called ‘sparsity factor’ variable [49]) and $q_{i}$ (called ‘quality factor’ variable [49]) expressed as follows

(18) $$s_{i}=\frac{S_{i}}{1-\gamma_{i}S_{i}}$$

(19) $$q_{i}=\frac{Q_{i}}{1-\gamma_{i}S_{i}}$$

(20) $$S_{i}=\beta \phi_{i}^{T}\phi_{i}-\beta^{2}\phi_{i}^{T}\Phi \Sigma \Phi^{T}\Phi_{i}$$

(21) $$Q_{i}=\beta \phi_{i}^{T}y-\beta^{2}\phi_{i}^{T}\Phi \Sigma \Phi^{T}y\;,$$

where Φ and Σ include only the columns that are selected in current iteration.

Suppose the sparsity of the shadowing image is *K*, the computational complexity of Laplace prior is $O(NK^{2})$ in the fast algorithm [48,50]. The fast realization when using *student-t* prior has the same computational complexity $O(NK^{2})$, however, as analyzed in [48], *student-t* prior has higher sparsity level than Laplace prior (i.e., $K_{student-t}>K_{Laplace}$). Therefore, the *student-t* prior requires more time than Laplace prior for target localization. For the Jeffrey’s prior, it demands intensive $O(N^{3})$ operations [51,52] to obtain the final solution.

The fast algorithm of RTI using SBL with Laplace prior is presented in Algorithm 1. To note, in practice, we find that the update of β is not necessary in each iteration, because the estimates at the early iterations are quite inaccurate, which deteriorate the quality of the shadowing images.

**Algorithm 1:** Fast algorithm of RTI using SBL with Laplace prior

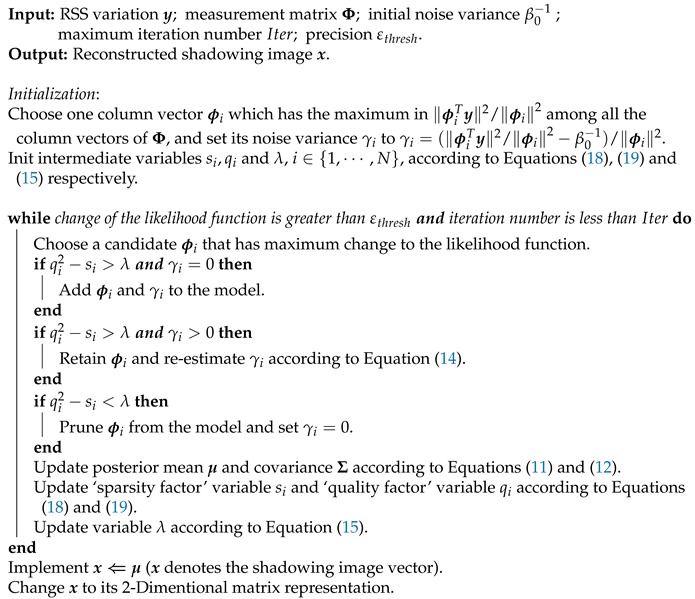



## 4. Experiments and Results

### 4.1. Experimental Setup

**Figure 4:**
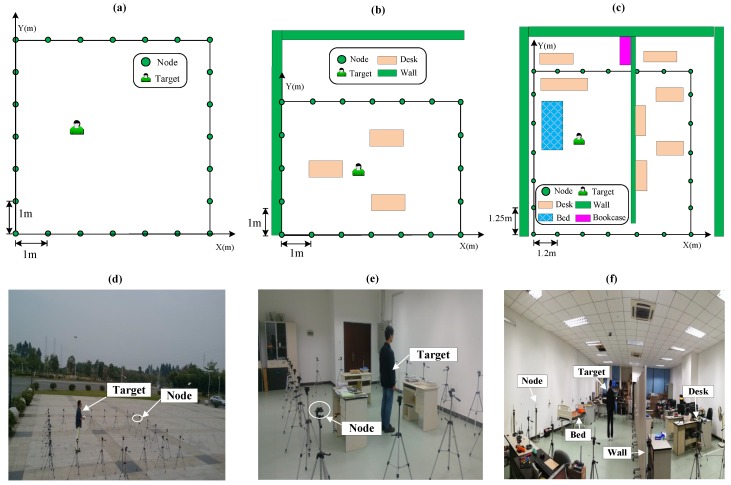
Experimental layouts and photos. (**a**,**d**) represent scenario 1. (**b**,**e**) denote scenario 2. (**c**,**f**) describe scenario 3.

The experiments are carried out in three different environments and Figure 4 illustrates the layouts and photos of the experiments. The MICAz made by Crossbow is taken as the RF node in the experiments. The pixel size is set equal to 0.2 m.

- Scenario 1: 24 RF-sensing nodes are deployed to cover an outdoor 6 m × 6 m area with each node 1 meter apart from the ground. The layout and photograph are presented in Figure 4a,d.
- Scenario 2: A sensing area of 6 m × 4 m is surrounded by 20 nodes in an indoor obstructed laboratory, as shown in Figure 4b,e. Three desks and some books are inside the sensing area.
- Scenario 3: The monitored 7.2 m × 7.5 m area is covered by 24 RF-sensing nodes in an indoor through-wall environment. As shown in Figure 4c,f, plenty of furniture, such as bed, desks, bookcase and computers obstruct the LOS of many RF links.

In the wireless network, each node is allocated its unique ID number. Like in many other studies about RTI [1,5,14,17,24,25,27], the low-cost narrow band IEEE 802.15.4 standard with 2.4 GHz frequency is used for communication. The low hardware cost renders the deployment of large and dense wireless network possible used for device-free target localization. Moreover, this dense deployment guarantees certain number of RF links are obstructed by the target and provides enough shadowing RSS measurements for target localization. Following the method in other studies about RTI, the token ring protocol is adopted to control the time-division sequence in the network. At one instant, only one node that catches the token can transmit the packet, other nodes are in the receiving state and store the received signal strength (RSS) in their memories. After the transmission, this node passes the token to the next node according to the pre-defined sequence. The link from the node to itself does not contain the informative shadowing RSS component used for target localization. Thus, the node is disabled to receive the RF signal from itself in our experimental settings.

**Figure 5:**
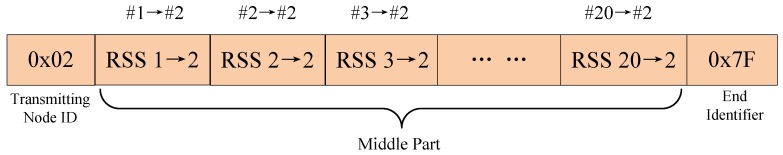
One illustrative packet transmitted by the second node in scenario 2.

The packet transmitted by each node contains three parts: ID of the transmitting node, RSS information that the packet carries in the middle part, and the end identifier. Figure 5 illustrates one example packet transmitted by the 2nd node in scenario 2. The start of the packet is the node number, which is assigned with 0x02 because the ID of the transmitting node is #2. The middle part of the packet comprises 20 bytes, because there are 20 nodes in scenario 2. Each byte in the middle part denotes the previously stored RSS received from other nodes when this node is in the receiving stage. Specifically, the first byte in the middle part represents the received RSS from node #1, the second byte representing RSS from itself (invalid and is assigned with zero decibel in practice), the third byte representing RSS from #3, and so forth, until the last byte denoting RSS received from node #20. The last part of the packet is 0x7F, which serves as the end identifier. The readers can appoint other characters as the end identifier in their experiments.

**Figure 6:**
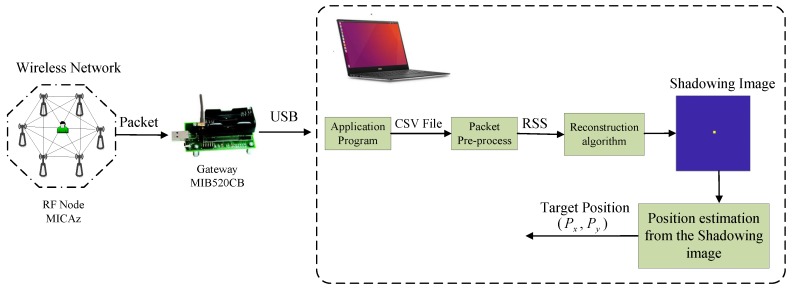
Framework of the experimental system.

In addition, there is an extra node outside the wireless network, which is MIB520CB made by Crowsbow in our experiment. This node plays the role of gateway which listens to the packets transmitted by each node in the wireless network and sends them to the computer via USB port. On the computer, an application program developed based on Visual C++ is used to read data from USB serial port and stores the packets to a file with CSV format. Then the CSV file is imported to MATLAB and pre-processed by a function of mean filter on MATLAB. Thereafter, the averaged RSS measurements are taken as the input to the reconstruction algorithm. After that, the shadowing image is obtained, which is then used to estimate the target’s location. The framework of the experimental system is presented in Figure 6.

In the following experiments, the Laplace prior is compared with *student-t* prior and Jeffrey’s prior. The robustness to multipath fading will be validated via imaging, localization and tracking experiments. The experimental evaluation parameters are given in Table 1.

### 4.2. Imaging Experiment

The robustness to multipath fading can be reflected on the improved image quality in RTI. Before the imaging experiment, a calibration procedure is performed as described in the 2nd paragraph of Section 2.1. In the experiments, the target stands at 66 different positions in scenario 1, 33 positions in scenario 2 and 30 positions in scenario 3. In each case, the shadowing image is reconstructed under Laplace prior, *student-t* prior and Jeffrey’s prior respectively based on SBL.

Inspired by the work in [1], the cylindrical model is used as the “true” shadowing image $x_{c}$ for reference, which is formulated as

(22) $$x_{cj}=\left\{\begin{array}{cc} 1 & if\;||P_{j}-P_{true}{\left|\right|}_{\ell_{2}}<R\\ 0 & otherwise \end{array}\right.,$$

where $x_{cj}$ is the pixel value of the *j*-th pixel in $x_{c}$. $P_{j}\;,\;P_{true}$ and *R* denote the center coordinate of the *j*-th pixel, the target’s true position and the radius of the cylinder respectively. Considering that the target has the waist width of about 0.76 m, thus for simplicity, *R* is set to 0.4 m in the following experiment. Moreover, the pixels that have non-zero pixel values outside the circular region are taken as artifacts.

**Figure 7:**
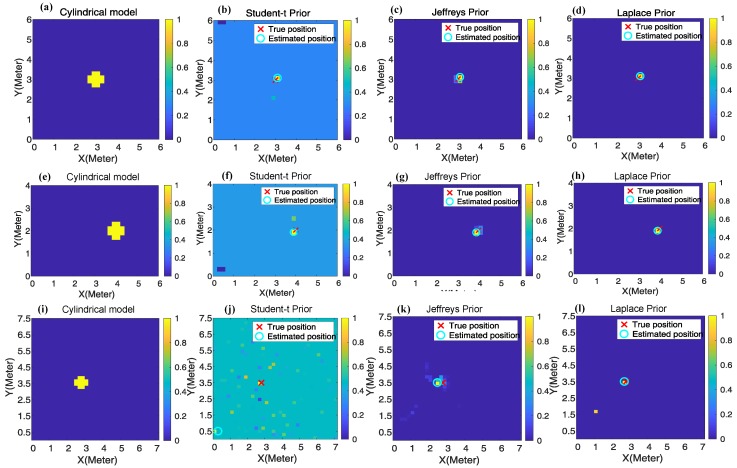
Images from the cylindrical model and Reconstructed images from different priors using SBL. (**a**–**d**) describe scenario 1. (**e**–**h**) illustrate the images in scenario 2. (**i**–**k**) demonstrate the images in scenario 3.

Figure 7 shows three groups of images. All the images are normalized and are vividly shown in the colorful illustration. Different color stands for different attenuation value, ranging from zero to one. The larger the attenuation-value (or pixel value) is, the more possible the target locates there. The top row, middle row and bottom row represent the images from scenarios 1–3 correspondingly. From left to right on each row, they denote the results from the cylindrical model, student-t prior, Jeffrey’s prior and Laplace prior respectively. As can be seen, both the Laplace prior and Jeffrey’s prior can reduce the artifacts in the reconstructed images compared to *student-t* prior, and Laplace prior achieves the best performance among them.

From an overall perspective, we record the number of artifacts contained in the reconstructed image in each experiment and compute the average as one metric for comparison. The results are shown in Table 2. As can be seen, Laplace prior could obtain the shadowing image with least artifacts and this advantage is more obvious in the cluttered scenario 3 compared with that in scenarios 1 and 2.

Following the method in [1], the MSE criterion defined in Equation (23) is also used to evaluate the quality of the reconstructed images

(23) $$\tau =\frac{\left|\right|\hat{x}-x_{c}{\left|\right|}_{\ell_{2}}^{2}}{N},$$

where *N* is the total number of pixels in the image. $\hat{x}$ and $x_{c}$ represent the vectorial representation of the normalized reconstructed image and the “true” shadowing image from the cylindrical model. Table 3 reports the comparative results of the reconstructed images in MSE. As can be seen, Laplace prior achieves the least MSE compared with Jeffrey’s prior and *student-t* prior. This result consists with the result shown in Table 2, which leads us to draw the conclusion that Laplace prior can improve the quality of the shadowing image via weakening the interference from multipath fading.

### 4.3. Device-free Localization Experiment

After the shadowing image $\hat{x}$ is obtained, the next step is to estimate the target’s position from the image. Usually the target is assumed to be located at the center coordinate of the pixel with maximum pixel value, which is described as follows

(24) $$\hat{P}=P_{k}=\left[P_{kx},P_{ky}\right]\;\;\;\;k=\underset{i}{\arg\max}{\hat{x}}_{i},$$

where $P_{k}$ denotes the central coordinate of the *k*-th pixel and ${\hat{x}}_{i}$ stands for the pixel value of the *i*-th pixel in the estimated shadowing image $\hat{x}$. The estimated position of the target is denoted as a cyan circle in each image of Figure 7. The localization error is defined as the Euclidean $\ell_{2}$ norm of the distance between the estimated position $\hat{P}$ and the true position $P_{true}$.

(25) $$e_{loc}={∥\hat{P}-P_{true}∥}_{\ell_{2}}$$

**Figure 8:**
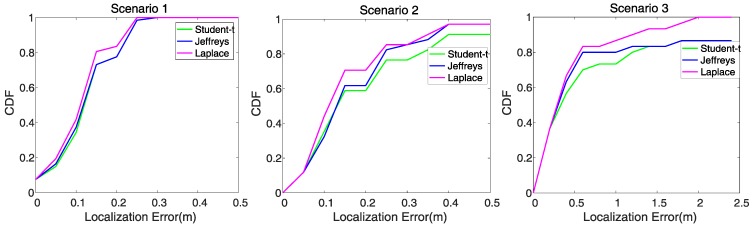
CDF of Localization errors (**a**) scenario 1 (**b**) scenario 2 (**c**) scenario 3.

In the device-free localization experiment, also the target stands at 66 different pre-defined positions in scenario 1, 33 positions in scenario 2 and 30 positions in scenario 3. In each position, the RSS variation $y=\bar{r}-r$ is computed as the observation for image reconstruction and target localization. Thereafter, the shadowing image is reconstructed using different priors based on SBL, and the target’s location and localization error are computed following Equations (24) and (25). The localization error is recorded in each localization experiment and we take the average for comparison between different priors. The results of the mean localization error are summarized in Table 4. Meanwhile, the CDF (Cumulative Distribution Function) curves of the localization errors in each scenario are shown in Figure 8.

As can be seen from Table 4 and Figure 8, both Laplace prior and Jeffrey’s prior have less localization errors than *student-t* prior, and Laplace prior is more advantageous, particularly in the cluttered scenario 3. The above results in localization also reveal that RTI using SBL with Laplace prior is robust to multipath fading.

### 4.4. Experiment in Localization Time

To validate the improved computational efficiency of using Laplace prior in localization, the time consumption experiment is conducted. As the same procedure described in Section 4.3, 66 different pre-defined positions in scenario 1, 33 positions in scenario 2 and 30 positions in scenario 3 are used as the samples. The localization algorithm is implemented on the platform of MATLAB. For each sample, the localization time (recorded by the CPU execution time) using different priors under SBL is recorded. Then the average time consumption is computed as the result for comparison, which is reported in Table 5. The results are obtained on a computer with 3.7 GHz CPU and 4G RAM.

As can be seen from Table 5, a localization system using Laplace prior can reduce the localization time compared with *student-t* prior and Jeffrey’s prior. Although Jeffrey’s prior has lower localization error as reported in Table 4, it demands much more time as illustrated in Table 5. By contrast, the localization system using Laplace prior under SBL can achieve better localization accuracy with lower time consumption as well.

### 4.5. Device-free Tracking Experiment

**Figure 9:**
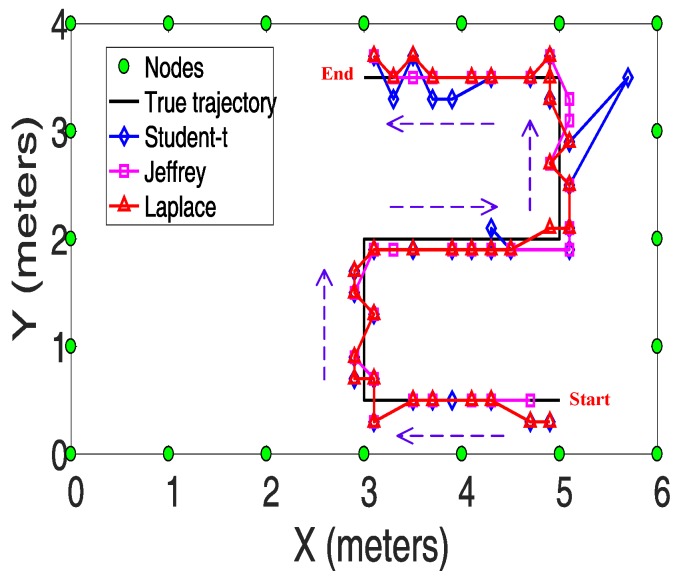
True trajectory and Estimated trajectories under different priors.

To further validate the effectiveness of the proposed Laplace prior in reducing the interference from multipath fading, we conduct the tracking experiment in scenario 2. Before the tracking experiment, the RSS measurements for all the links in the empty scenario are collected in advance, which is denoted by $\bar{r}$. Thereafter, as shown in Figure 9, the target moves from the start point to the end point following the directions as the arrows indicate. During this procedure, the RSS measurements are recorded which is denoted by r. Then we take $y=\bar{r}-r$ as the observation used for tracking.

In Figure 9, the estimated trajectory is connected by 36 position samples. Each position sample is obtained via the localization algorithm presented in Section 4.3. The mean tracking error is defined as the average of the localization errors from each position sample.

(26) $$e_{track}=\frac{1}{36}\sum_{i=1}^{36}{\left(e_{loc}\right)}_{i}$$

The mean tracking errors for different priors are reported in Table 6. As can be seen, the Laplace prior can achieve the least tracking error because of its robustness to multipath interference.

## 5. Conclusions and Future Research

To enhance the robustness to multipath fading in narrow-band RF localization, this paper explores the Laplace prior to characterize the shadowing image. Bayesian modeling and Bayesian inference especially the fast algorithm are presented to obtain the MAP solution in this paper. Experiments from three different scenarios are conducted to validate the advantages of the proposed approach in dealing with the multipath fading. Imaging, localization and tracking results reveal that RTI using SBL with Laplace prior is robust to multipath fading. Meanwhile, the proposed strategy improves the computational efficiency in localization as well.

Future research will be necessary to establish the prior that is suitable for multi-target case. Because each target may only occupy few pixels near the position where he stands, the whole image can be separated into several blocks or groups according equaling to the target number. Perhaps the structured priors could be explored to extend the proposed strategy to multi-target situation.

## Figures and Tables

**Table 1:** Experimental Evaluation Parameters.

Symbol	Appearance	Value	Explanation
δ	Section 2.1	0.2 m	Pixel size
η	Equation (2)	0.02 m	Ellipse width
ν	Equation (6)	2	Shape and scale parameters for λ
*c*	Equation (9)	1	Shape parameter for β
*d*	Equation (9)	0	Scale parameter for β
$\beta_{0}^{-1}$	Algorithm 1	0.01	Initial noise variance
Iter	Algorithm 1	1000	Maximum iteration number
$\varepsilon_{thresh}$	Algorithm 1	0.01	Precision during the iteration
*R*	Equation (22)	0.4 m	Radius of the cylindrical model

**Table 2:** Average number of artifacts in the reconstructed images.

Setup	*Student-t* Prior	Jeffrey’s Prior	Laplace Prior
Scenario-1	1.5	0.3	**0.2**
Scenario-2	2.2	1.2	**0.3**
Scenario-3	90.8	79.5	**1.1**

**Table 3:** Comparative results of the reconstructed images in MSE.

Setup	*Student-t* Prior	Jeffrey’s Prior	Laplace Prior
Scenario-1	0.0232	0.0024	**0.0023**
Scenario-2	0.1041	0.0182	**0.0085**
Scenario-3	0.1916	0.0042	**0.0018**

**Table 4:** Comparison of the mean localization errors (unit: m).

Setup	*Student-t* Prior	Jeffrey’s Prior	Laplace Prior
Scenario-1	0.13	0.12	**0.11**
Scenario-2	0.33	0.19	**0.18**
Scenario-3	0.88	0.70	**0.43**

**Table 5:** Average time consumption in localization (unit: ms).

Setup	*Student-t* Prior	Jeffrey’s Prior	Laplace Prior
Scenario-1	30.3	749.9	**27.2**
Scenario-2	21.3	449.1	**15.1**
Scenario-3	39.8	2802.5	**30.2**

**Table 6:** Tracking error comparison (unit: m).

Prior	Tracking Error
*Student-t*	0.26
Jeffrey’s	0.23
Laplace	0.21

## Abbreviations

The following abbreviations are used in this manuscript:

RTI	Radio Tomographic Imaging
RF	Radio Frequency
RSS	Received Signal Strength
LOS	Line of Sight
NLOS	Non Line of Sight
SBL	Sparse Bayesian Learning
MAP	maximum-a-posterior
PIR	pyroelectric infrared
UWB	ultra-wide band
CSI	Channel State Information
SVD	Singular Value Decomposition
HBCS	heterogeneous Bayesian compressive sensing
MSE	Mean Square Error
CDF	Cumulative Distribution Function
**Variable List**	
**Variable**	**Explanation**
y	RSS variation
x	Vector of the shadowing image
n	Measurement noise
Φ	Measurement matrix
dis	Distance between the transmitter and the receiver
$d_{i,j}^{TX}$	Distance between the target and the transmitter
$d_{i,j}^{RX}$	Distance between the target and the receiver
λ	Scale parameter of the Laplace distribution
γ	Vector of the signal variance
β	Inverse of the noise variance
μ	Posterior mean
Σ	Posterior covariance
$s_{i}$	‘Sparsity factor’ variable
$q_{i}$	‘Quality factor’ variable
$\hat{x}$	Vector of the estimated shadowing image
$x_{c}$	Vector of the reference shadowing image
τ	Mean square error of the shadowing image
$\hat{P}$	Estimated position of the target
$P_{true}$	True position of the target
$e_{loc}$	Localization error
$e_{track}$	Mean tracking error

## References

[1] Joey W., Neal P. (2010). Radio tomographic imaging with wireless networks. IEEE Trans. Mob. Comput..

[2] Dustin M., Joey W., Neal P. Toward a rapidly deployable radio tomographic imaging system for tactical operations. Proceedings of the 38th Annual IEEE Conference on Local Computer Networks.

[3] Cesare A., Maurizio B., Giacomo B., Neal P., Manuel R. (2016). RTI goes wild: Radio tomographic imaging for outdoor people detection and localization. IEEE Trans. Mob. Comput..

[4] Christopher A., Martin R., Owens W., Ryan T. (2014). Radio tomography for roadside surveillance. IEEE J. Sel. Top. Signal Process..

[5] Ossi K., Maurizio B., Neal P. Follow@ grandma: Long-term device-free localization for residential monitoring. Proceedings of the 37th Annual IEEE Conference on Local Computer Networks.

[6] Nathavuth K., Hirozumi Y., Teruo H. (2019). EasyTrack: Zero-Calibration Smart-Home Tracking System. J. Inf. Process. Syst..

[7] Maurizio B., Ossi K., Neal P. (2013). Radio Tomographic Imaging for Ambient Assisted Living. Evaluating AAL Systems Through Competitive Benchmarking.

[8] Goncalo M., Rui P. (2016). An Indoor Monitoring System for Ambient Assisted Living Based on Internet of Things Architecture. Int. J. Environ. Res. Public Health.

[9] Millar G., Aghdasi F., Lei W. (2017). Tracking Moving Objects using a Camera Network. U.S. Patent.

[10] Jong-Wan Y., Taejoon P. (2016). Maximizing Localization Accuracy via Self-Configurable Ultrasonic Sensor Grouping Using Genetic Approach. IEEE Trans. Instrum. Meas..

[11] Zhang S., Liu K.H., Ma Y.T., Huang X.D., Gong X.L., Zhang Y.L. (2018). An Accurate Geometrical Multi-Target Device-Free Localization Method Using Light Sensors. IEEE Sens. J..

[12] Jurgen K., Daniel H. Passive infrared localization with a Probability Hypothesis Density filter. Proceedings of the 7th Workshop on Positioning, Navigation and Communication.

[13] Sujay N., Sujay N., Vijay S.R., Prabhakar T.V., Sripad S.K., Madhuri S.I. (2015). PIR sensors: Characterization and novel localization technique. IPSN ’15 Proceedings of the 14th International Conference on Information Processing in Sensor Networks.

[14] Wilson J., Patwari N. (2011). See-through walls: Motion tracking using variance-based radio tomography networks. IEEE Trans. Mob. Comput..

[15] Wang X.Y., Gao L.J., Mao S.W., Santosh P. (2017). CSI-based fingerprinting for indoor localization: A deep learning approach. IEEE Trans. Veh. Technol..

[16] Wu K., Xiao J., Yi Y., Chen D., Luo X., Ni L. (2013). CSI-based indoor localization. IEEE Trans. Parallel Distrib. Syst..

[17] Neal P., Joey W. (2010). RF Sensor Networks for Device-Free Localization: Measurements, Models, and Algorithms. Proc. IEEE.

[18] Henri N., Anssi R., Simo A.L., Robert P. Particle filter and smoother for indoor localization. Proceedings of the International Conference on Indoor Positioning and Indoor Navigation.

[19] Min P.J., Ki A.C., Yuriy S.S., Peng S., Taeg L.M. (2017). Accurate and reliable human localization using composite particle/FIR filtering. IEEE Trans. Hum.-Mach. Syst..

[20] Li Z., Liu J.B., Yang F., Niu X.G., Li L.L., Wang Z.M., Chen R.Z. (2018). A Bayesian Density Model Based Radio Signal Fingerprinting Positioning Method for Enhanced Usability. Sensors.

[21] Erick S., Misbahuddin A.M., David A. (2018). A Performance Study of a Fast-Rate WLAN Fingerprint Measurement Collection Method. IEEE Trans. Instrum. Meas..

[22] Talampas M.C.R., Low K.S. (2016). A geometric filter algorithm for robust device-free localization in wireless networks. IEEE Trans. Ind. Inf..

[23] Zhang J., Xiao W.D., Zhang S., Huang S.D. (2017). Device-Free Localization via an Extreme Learning Machine with Parameterized Geometrical Feature Extraction. Sensors.

[24] Kaltiokallio O., Jäntti R., Patwari N. (2017). ARTI: An Adaptive Radio Tomographic Imaging System. IEEE Trans. Veh. Technol..

[25] Huseyin Y., Riku J., Ossi K., Neal P. (2017). Detector Based Radio Tomographic Imaging. IEEE Trans. Mob. Comput..

[26] Wang Z., Su H., Guo X.M., Wang G.L. Radio Tomographic Imaging with Feedback-Based Sparse Bayesian Learning. Proceedings of the 2018 Eighth International Conference on Information Science and Technology (ICIST).

[27] Daniel R., Donghoon L., Georgios B.G. (2018). Blind Radio Tomography. IEEE Trans. Signal Process..

[28] Yigitler H. (2018). Narrowband Radio Frequency Inference: Physical Modeling and Measurement Processing. Ph.D. Thesis.

[29] Yigitler H., Ossi K., Riku J. (2018). Received Signal Strength Models for Narrowband Radios.

[30] Jakub N., Zdenek T., Vlastimil B., Ladislav P., Ondrej K., Libor B., Jiri S., Tomas K. Study of the performance of RSSI based Bluetooth Smart indoor positioning. Proceedings of the 2016 26th International Conference Radioelektronika (RADIOELEKTRONIKA).

[31] Bo W., Ambuj V., Neal P., Wen H., Thiemo V., Chou C.T. dRTI: Directional radio tomographic imaging. Proceedings of the 14th International Conference on Information Processing in Sensor Networks.

[32] Cheng Q., Peter H., Amal A.H., Neal P., Gregory D.D. (2017). On-Wall, Wide Bandwidth E-Shaped Patch Antenna for Improved Whole-Home Radio Tomography. IEEE J. Radio Freq. Identif. (RFID).

[33] Xu S.X., Liu H., Gao F., Wang Z.H. (2019). Compressive Sensing Based Radio Tomographic Imaging with Spatial Diversity. Sensors.

[34] Kaltiokallio O., Bocca M., Patwari N. Enhancing the accuracy of radio tomographic imaging using channel diversity. Proceedings of the 2012 IEEE 9th International Conference on Mobile Ad-Hoc and Sensor Systems (MASS 2012).

[35] Stijn D., Rafael B., Glenn E., Maarten W. Multi-frequency sub-1 GHz radio tomographic imaging in a complex indoor environment. Proceedings of the 2017 International Conference on Indoor Positioning and Indoor Navigation (IPIN).

[36] Jin J., Ke W., Lu J., Wang Y.L., Zoran S. Multi-channel RTI fusion based on improved joint sparse model. Proceedings of the 2018 Ubiquitous Positioning, Indoor Navigation and Location-Based Services (UPINLBS).

[37] Wang J., Gao Q., Wang H., Cheng P., Xin K. (2015). Device-free localization with multidimensional wireless link information. IEEE Trans. Veh. Technol..

[38] Wilson J., Patwari N. (2012). A fade-level skew-laplace signal strength model for device-free localization with wireless networks. IEEE Trans. Mob. Comput..

[39] Yang L.W., Huang K.D., Wang G.L., Guo X.M. An enhanced multi-scale model for shadow fading in radio tomographic imaging. Proceedings of the 11th World Congress on Intelligent Control and Automation.

[40] Tan J.J., Zhao X., Yang L.W., Guo X.M., Wang G.L. Backprojection and Integration for the Multi-Scale Spatial Model in Radio Tomographic Imaging. Proceedings of the 2018 IEEE 8th Annual International Conference on CYBER Technology in Autoumation, Control, and Intelligent System (CYBER).

[41] Tan J.J., Zhao Q.Q., Guo X.M., Zhao X., Wang G.L. (2019). Radio Tomographic Imaging Based on Low-Rank and Sparse Decomposition. IEEE Access.

[42] Huang K.D., Guo Y., Guo X.M., Wang G.L. (2014). Heterogeneous Bayesian compressive sensing for sparse signal recovery. IET Signal Process..

[43] Huang K.D., Tan S.B., Luo Y.B., Guo X.M., Wang G.L. (2017). Enhanced radio tomographic imaging with heterogeneous Bayesian compressive sensing. Pervasive Mob. Comput..

[44] Andrea E., Michael R. (2013). Background Subtraction for Online Calibration of Baseline RSS in RF Sensing Networks. IEEE Trans. Mob. Comput..

[45] Viani F., Rocca P., Oliveri G., Trinchero D., Massa A. (2011). Localization, tracking, and imaging of targets in wireless sensor networks: An invited review. Radio Sci..

[46] Tipping M.E. (2001). Sparse Bayesian learning and the relevance vector machine. J. Mach. Learn. Res..

[47] Wang Z., Qin L., Guo X.M., Wang G.L. (2019). Dual radio tomographic imaging with shadowing-measurement awareness. IEEE Trans. Instrum. Meas..

[48] Derin B.S., Rafael M., Aggelos K.K. (2010). Bayesian Compressive Sensing Using Laplace Priors. IEEE Trans. Image Process..

[49] Michael E.T., Anita F. Fast Marginal Likelihood Maximisation for Sparse Bayesian Models. Proceedings of the Ninth International Workshop on Artificial Intelligence and Statistics (AISTATS).

[50] Ji S.H., Xue Y., Lawrence C. (2008). Bayesian Compressive Sensing. IEEE Trans. Signal Process..

[51] Figueiredo M. Adaptive sparseness using Jeffreys prior. Proceedings of the 14th International Conference on Neural Information Processing Systems: Natural and Synthetic.

[52] Kun M. Jeffreys Prior: Philosophy, Information Geometry and Empirical Bayesian Methods. https://www.researchgate.net/publication/323736141_Jeffreys_Prior_Philosophy_Information_Geometry_and_Empirical_Bayesian_Methods.

